# Case report: BCR-ABL-positive acute lymphoblastic leukemia with bone destruction: a treatment dilemma

**DOI:** 10.3389/fonc.2024.1356311

**Published:** 2024-02-21

**Authors:** Shi Lijun, Ma Zhongrui, Wei Li, Yu Xia, Jiang Wei, Pan Yaning

**Affiliations:** ^1^ Department of Hematology, Chengdu Fifth People’s Hospital, Chengdu, China; ^2^ Department of Psychosomatic Medicine, Chengdu Fifth People’s Hospital, Chengdu, China; ^3^ Geriatric Diseases Institute of Chengdu, Chengdu, China

**Keywords:** acute lymphoblastic leukemia, hypercalcemia, osteolysis, osteolytic bone lesions, protein kinase inhibitors

## Abstract

Although bone destruction and hypercalcemia without acute peripheral blast BCR-ABL-positive acute lymphoblastic leukemia (ALL) have been reported in children, they are rare in adults. Herein, we describe a case of BCR-ABL positive ALL with a triploid karyotype, WT1, and CDKN2A mutations with hypercalcemia and bone destruction as the first manifestations. Complete remission (CR) was achieved by induction chemotherapy. BCR-ABL turned negative after treatment with dasatinib. However, computed tomography and whole-body bone scan showed extensive bone destruction. Additionally, bone biopsy showed leukemic infiltration. After treatment with dasatinib and VMCP, leukemia recurred with positive BCR-ABL. The T315I mutation occurred. The patient was surgically diagnosed with calculous cholecystitis and achieved CR2 by postoperative orebatinib and VP regimens. Later, the patient died due to a severe pulmonary infection. BCR-ABL-positive ALL with bone destruction is rare and difficult to control using tyrosine kinase inhibitor chemotherapy alone. Therefore, further exploration of more effective treatments is needed.

## 1 Introduction

BCR-ABL-positive acute lymphocyte leukemia (ALL) accounts for approximately 20% of ALL cases in adults (1). Its clinical manifestations are the same as those of other ALL cases, including infection, hemorrhage, anemia, lymphadenopathy, hepatosplenomegaly, and central nervous system infiltration. Bone destruction and hypercalcemia are rare in ALL, except for in adult T-cell leukemia/lymphoma (ATLL) (2). Few case reports on osteolytic lesions have been published. Furthermore, the prognostic significance of bone damage remains unclear (3). Although similar cases have been reported, these were all BCR-ABL-negative ALL. Herein, we report the diagnosis and treatment of a patient with BCR-ABL-positive ALL with bone destruction and secondary hypercalcemia as the first manifestations and also review the relevant literature. This condition is extremely rarely encountered in clinical practice. Therefore, it needs to be reported to aid clinicians’ decision-making regarding diagnosis and treatment.

## 2 Case description

**Figure 1:**
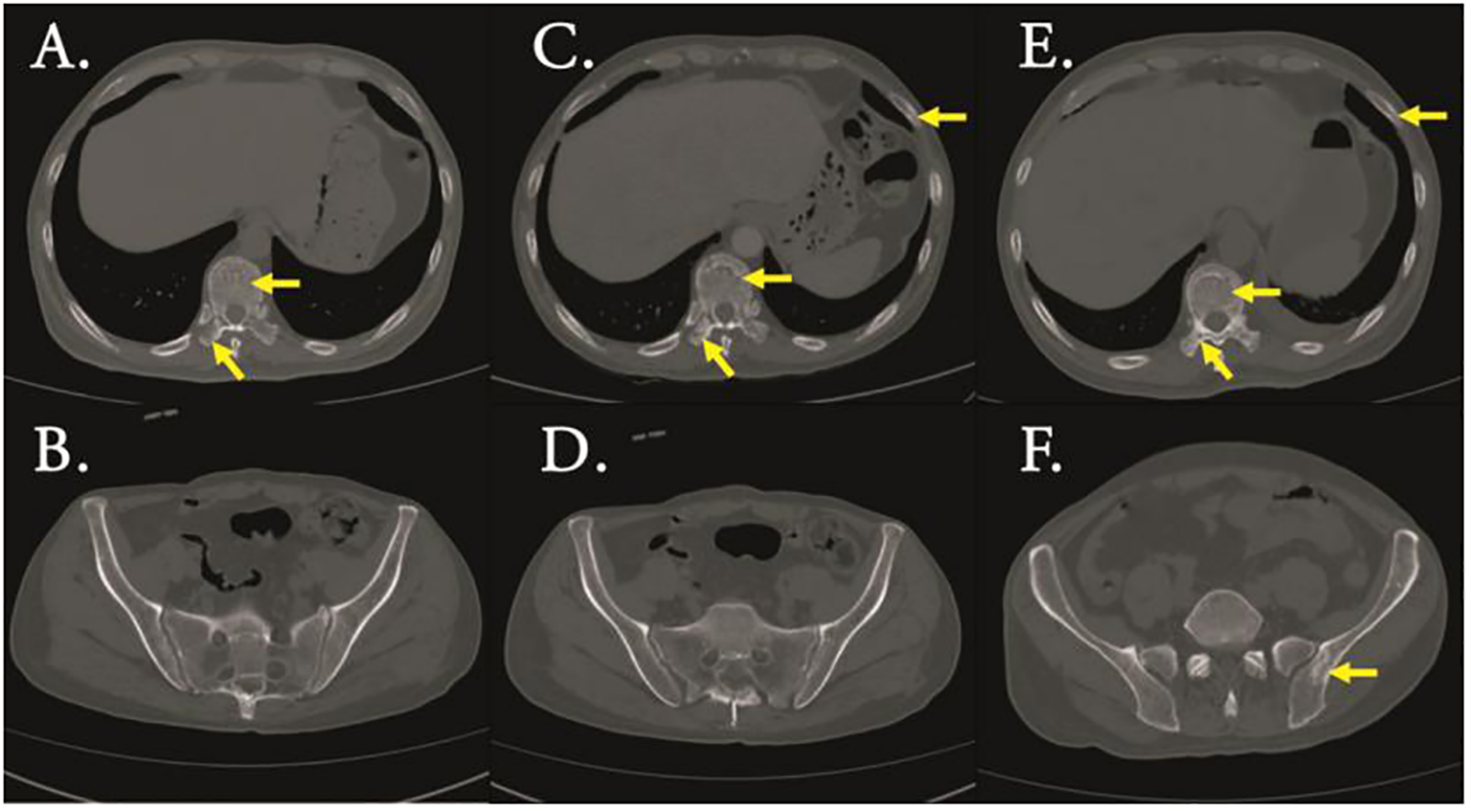
CT scan (bone window) revealed bone destruction of Thoracic vertebrae and transverse processes **(A)** and ilium **(B)** of initial diagnosis,bone destruction of Thoracic vertebrae, transverse processes and rib **(C)** and ilium **(D)** of complete remission(CR),bone destruction of Thoracic vertebrae, transverse processes and rib **(E)** and ilium **(F)** of relapse.

**Figure 2:**
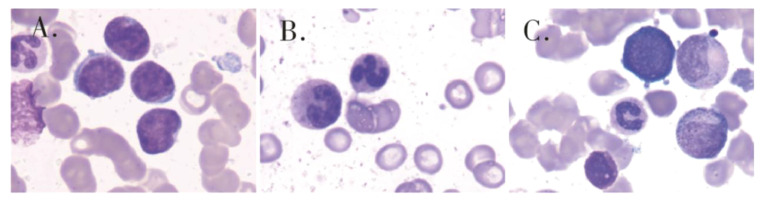
Bone marrow cytology showed that primitive immature lymphocytes accounted for 83% of cells **(A)** of initial diagnosis,1.5% of cells **(B)** of after one course of induction therapy and 50.5% of cells **(C)** of after two sessions of consolidation therapy.

**Figure 3:**
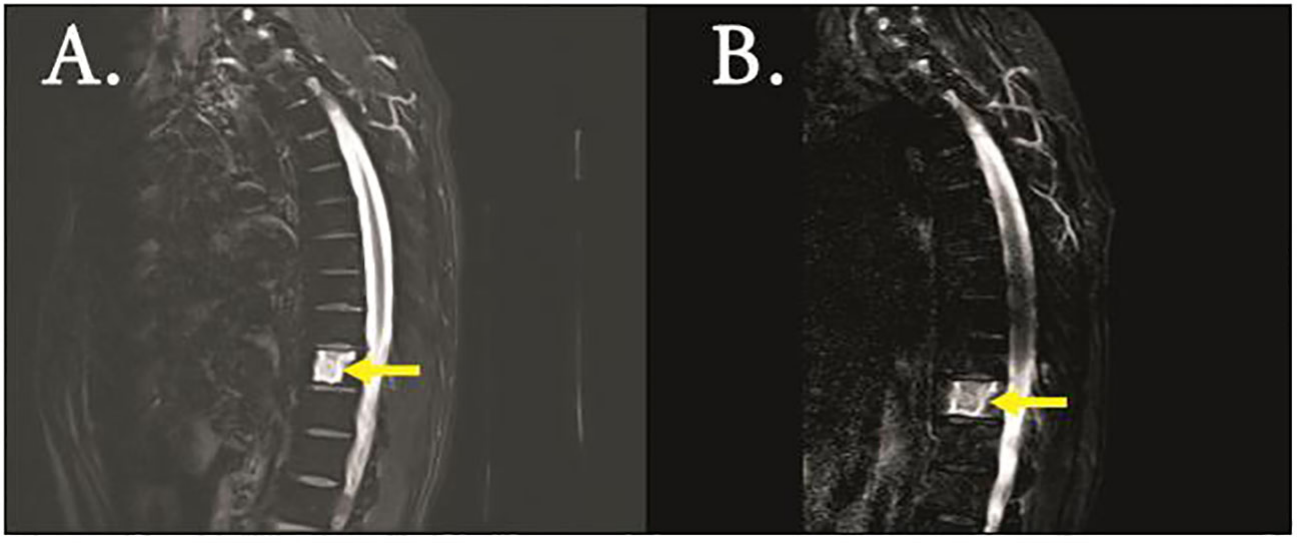
MRI (T2 imaging) revealed destruction of the thoracic vertebrae of initial diagnosis **(A)** and CR **(B)**.

**Figure 4:**
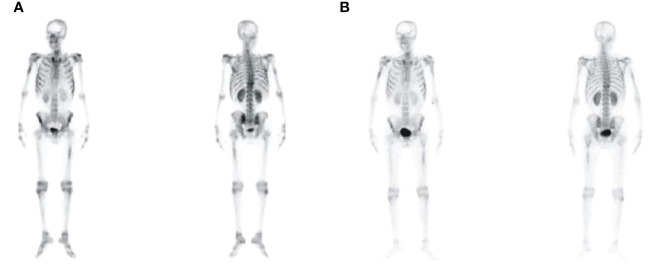
Bone imaging revealed multiple lesions of increased bone metabolism throughout the body of CR1 **(A)** and CR2 **(B)**.

**Figure 5:**
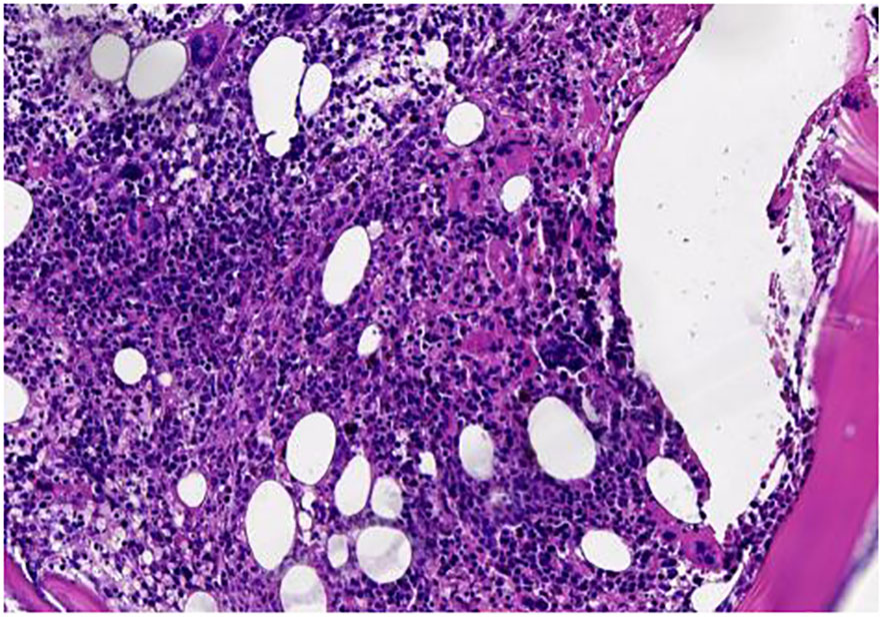
T10 vertebral bone biopsy (HE staining, 20-fold).

A 47-year-old male patient was admitted to the hospital with lower back pain for more than 3 months, which had worsened with a subcutaneous hemorrhage for 1 day. On June 23, 2022, the patient developed lower back pain without an obvious cause, accompanied by intermittent rib pain that was previously untreated. In July 2022, the pain worsened, and activities of daily living were limited. Computed tomography (CT) performed at another hospital showed focal bone destruction in the thoracic vertebrae and right transverse process (**Figure 1**). Routine blood tests at that time showed a white blood cell (WBC) count, 9.8 × 10^9^/L; hemoglobin (Hb) level, 142 g/L; and platelet (Plt) count, 106 × 10^9^/L. After symptomatic analgesic treatment, the symptoms were relieved but soon aggravated again. Moreover, a subcutaneous hemorrhage occurred 1 day before admission. The laboratory test results revealed a WBC count of 14.5 × 10^9^/L, 41% primitive blast cells, Hb level, 139 g/L, Plt count 7.0 × 10^9^/L, and normal renal function. The serum calcium level was 3.0 mmol/L (2.17–2.54). Bone marrow cytology showed that primitive immature lymphocytes accounted for 83% of cells (**Figure 2**). Flow cytometry of bone marrow cells showed that blast cells accounted for approximately 37.6% of the total nuclear cells and expressed cluster of differentiation (CD)10, CD19, CD34, and HLA-DR. They were negative for CD2, CD3, CD5, CD7, CD8, CD11b, CD13, CD14, CD15, CD16, CD20, CD33, CD56, CD64, and CD117, consistent with the immunophenotype of B-cell ALL. The karyotype was 76 (3n), XX, -Y, +2, +4, +6, -7, +8, t (9:22) (q34.1:q11.2), +13, -15, +16, +17, +20, +21, +22 (4)/46, XY (5). Leukemia fusion gene detection revealed BCR-ABL1 (P190) positivity. The WT1 mutation, CDKN2A (chr9p21.3) copy number deletion (NM_058195:exon 1–3/NM_000077:exon 1–3, length approximately 26 kb), and CDKN2B (chr9p21.3) (NM_004936:exon 2, length approximately 359 bp). The patient was diagnosed with BCR-ABL-positive (P190) B-ALL (a high-risk group with WT1 and CDKN2A mutations and a triploid karyotype) and hypercalcemia. After treatment with salmon calcitonin at the local hospital, the patient was administered VDCLP regimen on September 20, 2022 (vindesine 2.8 mg/m^2^, days 1, 8, 15, and 22; daunorubicin 40 mg/m^2^, days 1–3; cyclophosphamide 750 mg/m^2^, days 1 and 15; pegaspargase 3,750 U, days 1 and 15; dexamethasone 10 mg, days 1–14; and tapered off on day 15). Severe pulmonary infection occurred during chemotherapy. Therefore, the patient was transferred to our hospital on October 18, 2022, after receiving anti-infection treatment. Routine blood test at admission showed a WBC count, 8.5 × 10^9^/L; Hb level, 73 g/L; and Plt count, 389 × 10^9^/L. The serum calcium level was 1.9 mmol/L. Chest CT revealed scattered infection in both lungs, focal bone destruction in the thoracic vertebrae and right transverse process, and uneven bilateral bone density in multiple ribs (**Figure 1**). Magnetic resonance imaging of the thoracic spine revealed osteopenia, an abnormal signal in the T10 vertebral body and its adnexa, and the T9 adnexa. Combined with the patient’s medical history, it was surmised that the leukemic infiltration involved the vertebral body as well as localized endplate defects at the upper edge of the L3 vertebral body, the upper and lower edges of the L4 vertebral body, and the upper edge of the L5 vertebral body (**Figure 3**). Bone marrow re-examination on October 20, 2022 revealed complete remission (CR), and the proportion of blast lymphocytes was approximately 1.5% (**Figure 2**). After admission, the patient was administered anti-infective treatment with cefoperazone-sulbactam, voriconazole, amphotericin, and caspofungin. However, the patient still had recurrent fever and cough. Fiberoptic bronchoalveolar lavage and NGS were performed for *P. carinii* infection. Dasatinib, 100 mg q.d., combined with a VP regimen (vindesine 4 mg, dexamethasone 10 mg, days 1–4, 8–11, 15–16, and 21–24) was administered on November 3, 2022. Bone marrow cytology re-examination after chemotherapy showed CR (proportion of naïve lymphocytes). The BCR-ABL1 (P190) test was negative. On December 9, cerebrospinal fluid (CSF) common, biochemical, and CSF flow cell immunotyping were performed, and no abnormalities were found; chemotherapy drugs (methotrexate 50 mg plus cytarabine 30 mg plus dexamethasone 5 mg) were also injected intrathecally to prevent central nervous system leukemia. However, pain persisted in the sternum, ribs, and lumbar spine, and tramadol hydrochloride was required for pain relief. Whole-body bone imaging revealed multiple foci of increased bone metabolism throughout the body, and extensive bone infiltration by hematologically malignant tumors was suspected (**Figure 4**). A bone biopsy of the T10 vertebral body revealed dysplastic lymphocytes with obvious fibrosis. Immunohistochemistry showed that dysplastic lymphocytes were sometimes positive for CD20, CD3, CD79a, TDT, CD99, CD7, MPO, PAX-5, and Ki-67 (approximately 3%–5%), consistent with the B-ALL cell phenotype and supported leukemic cell infiltration (**Figure 5**). Serum protein electrophoresis revealed an M protein band of 2.4%, with content of 1.39 g/L. The serum-free light chain combination consisted of kappa light chain (25.28 mg/L) and free lambda (36.34 mg/L). Serum immunofixation electrophoresis revealed IgG-k type. No abnormalities were observed in the urinary free light chain assemblies, and no M protein was detected using urinary protein electrophoresis. The presence of M proteins in patients’ peripheral blood is considered to be related to B lymphocytes or the immune response to antigens encountered during infection (6).

Based on the above examination, the patient’s multiple bone destructions were considered to be caused by leukemic cell infiltration. On January 13, 2023, the patient was administered dasatinib 100 mg q.d. combined with VMCP (vindesine 2.8 mg/m^2^, days 1, 8, 15, and 22; mitoxantrone liposomes 30 mg on day 1; 20 mg on day 8; cyclophosphamide 750 mg/m^2^ on days 1 and 8; prednisone 30 mg, twice daily days 1–14, tapered off on day 15). He was discharged on February 3, 2023 and continued dasatinib administration (100 mg q.d.) outside the hospital. However, he did not return to the hospital on time for the next course of chemotherapy. On March 6, 2023, he was admitted again because of abdominal pain. Multiple liver function tests showed that gamma-glutamyltransferase (GGT) and alkaline phosphatase (ALP) gradually increased. The patient’s GGT increased to 5,024 U/L (the normal range in our hospital was 8–50 U/L) and ALP to 1,298 U/L (the normal range in our hospital was 40–150 U/L). CT suggested multiple gallbladder stones with infection and bone destruction in the ribs, transverse processes, and ilium (**Figure 1**). Anti-infective treatments were administered. Re-examination of bone marrow cytology and flow cytometric immunophenotyping showed disease recurrence; the proportion of primitive naïve lymphocytes was 50.5% (**Figure 2**), BCR-ABL was positive, and the BCR-ABL (P190) gene copy number was 561,000. BCR-ABL-positive ALL relapsed with calculous cholecystitis; cholecystectomy was performed. ABL kinase domain mutation analysis revealed a T315I mutation, the number of circulating tumor cells in peripheral blood was 8, and the stromal type coefficient was 72.97%. Therefore, on April 6, 2023, an orebatinib and VP regimen was initiated (orebatinib 40 mg, q.d.; vindesine 2.8 mg/m^2^, days 1, 8, 15, and 22; prednisone 30 mg, b.i.d. days 1–4, 8–11, 15–18, and 22–25). On May 6, bone marrow CR showed 0% primitive, naive lymphocytes, BCR-ABL (P190) was negative, and bone destruction improved (**Figure 3**). However, GGT and ALP increased significantly, accompanied by abdominal pain. After dexamethasone treatment, GGT and ALP levels decreased, abdominal pain was relieved, and the patient continued one course of orebatinib and VP regimen. Unfortunately, the patient stopped allogeneic hematopoietic stem cell transplantation, took orebatinib orally by himself outside the hospital, and did not return to the hospital regularly for chemotherapy. In August 2023, the patient died of a secondary severe lung infection.

## 3 Discussion

ALL is a heterogeneous disease with a bimodal distribution of age of onset, most commonly seen in children aged 1–4 years and adults over 60 years (7, 8). More than 20% of children with ALL have bone pain at the onset, higher than that in adults with ALL. However, multiple bone destruction is rare in both children and adults with ALL (4, 9, 10). Among adult malignant tumors, multiple bone destruction incidence is 27%–35% in lung cancer, 25%–30% in breast cancer, 7%–30% in multiple myeloma, < 10% in malignant lymphoma, and < 1% in leukemia (2). The patient in this case report first experienced back pain, gradually progressed to multiple bone destructions, and was finally diagnosed after the appearance of peripheral primitive and immature lymphocytes. Although a few similar cases have been reported, the cases were BCR-ABL-negative ALL (2, 4, 8, 11), different from that reported in this study, making this case extremely rare.

The imaging manifestations of bone destruction in leukemia include osteopenia, a radiolucent zone of the metaphyseal, wormlike changes, periosteal reaction, osteolytic bone destruction, punctures, and vertebral collapse or sclerosis. Literature reports that 41%–70% of pediatric patients with ALL had radiographic bone lesions at diagnosis. However, the mechanisms underlying multiple bone destruction and hypercalcemia in leukemia remain unclear (11). This may be related to leukemic cell infiltration and direct destruction of bone tissue and changes in cytokine levels or hormones related to bone metabolism, including parathyroid hormone, vitamin D steroid, prostaglandin E2 (PGE2), tumor necrosis factor-α (TNF), interleukin (IL)-1, IL-2, IL-6, etc. (5, 9, 10, 12). Bone destruction in patients with B-ALL may also be related to receptor activators of nuclear factor κB ligand (RANKL) and PTHrP (13). RANKL is a member of the TNF superfamily. It cooperates with macrophage colony-stimulating factor (M-CSF) and immunoreceptor tyrosine-activating motif (ITAM)-mediated co-stimulation signals to activate a wide range of signaling cascades and downstream transcriptional regulators to drive osteoclast formation. PTHrP promotes bone resorption by regulating calcium and phosphorus metabolism in renal tubular epithelial cells, promoting the production of renal-derived vitamin D, and stimulating osteoclast activity (13). However, higher serum PTHrP levels were not detected in patients with leukemia, suggesting that the mechanism of bone destruction in leukemia is complex and needs to be confirmed in further studies. Unfortunately, we did not monitor 25(OH)-vitamin D, calcitonin, and PTH-related protein (PTHrP) levels in this patient. Moreover, whether bone destruction adversely affects ALL prognosis remains controversial (2, 8–10, 12).

There is no standard treatment for BCR/ABL positive ALL. The advent of tyrosine kinase inhibitors (TKIs) has significantly improved the survival of patients with BCR-ABL-positive ALL. However, it is well established that patients with BCR-ABL-positive ALL with additional chromosomal abnormalities have a poor prognosis (14). The patient, in this case, achieved CR after one course of standard adult ALL induction regimen (VDCLP), and BCR-ABL turned negative after one course of dasatinib + VP regimen. However, bone destruction further spread. Biopsy confirmed that the bone destruction was caused by leukemic cell infiltration. The patient was re-treated with dasatinib combined with a liposome chemotherapy regimen. However, leukemia recurred, and a T315I mutation occurred. After switching to third-generation orebatinib, CR was reached 1 month later, BCR-ABL turned negative again, and bone imaging suggested that multiple bone destruction improved, suggesting that in patients with BCR-ABL-positive ALL with multiple bone destruction, simple second-generation TKI combined with chemotherapy cannot effectively solve bone destruction lesions, which has been reported in previous studies (9). Third-generation TKI combined with chemotherapy may have a certain effect on extramedullary bone destruction. Sasaki K et al. reported that orebatinib, a third-generation TKI, has shown clinical efficacy in the treatment of resistant Ph-positive ALL, especially in patients carrying the T315I mutation (14). However, BCR-ABL became positive again 4 months after TKI treatment, and the patient relapsed with a T315I mutation, which is not easily explained. Whether relapse in this patient was related to more additional chromosomes or WT1 and CDKN2A mutations remains unclear. However, why bone destruction worsens when bone marrow reaches CR and BCR-ABL turns negative during dasatinib treatment cannot be reasonably explained. Further research is warranted to elucidate whether patients with BCR-ABL-positive ALL with extensive bone destruction have unique biological properties. Whether the chemotherapy intensity or second-generation TKI is insufficient to control the growth of leukemia cells is unclear. Therefore, in patients with BCR-ABL-positive ALL with multiple bone destruction as the main manifestation, whether direct application of third-generation TKI during the initial induction requires further assessment. However, a lasting effect could not be predicted. Therefore, the patient was actively prepared for allogeneic hematopoietic stem cell transplantation.

This article reports a rare case of BCR-ABL-positive ALL with multiple bone destruction as the first manifestation, which raises several questions regarding the diagnosis and treatment processes, including rapid loss of response and progression, ABL kinase domain mutation within a short period of time, bone marrow CR, BCR-ABL-negative conversion, and extramedullary disease. However, these are currently unexplained, and additional clinical data and mechanistic studies are required.

## Data availability statement

The original contributions presented in the study are included in the article/supplementary material. Further inquiries can be directed to the corresponding author.

## Ethics statement

Written informed consent was obtained from the individual(s) for the publication of any potentially identifiable images or data included in this article.

## Author contributions

SL: Conceptualization, Data curation, Formal analysis, Funding acquisition, Investigation, Methodology, Project administration, Resources, Software, Supervision, Validation, Visualization, Writing – original draft, Writing – review & editing. MZ: Conceptualization, Data curation, Formal analysis, Funding acquisition, Investigation, Methodology, Project administration, Software, Supervision, Validation, Writing – original draft. WL: Conceptualization, Data curation, Formal analysis, Investigation, Methodology, Project administration, Supervision, Validation, Writing – review & editing. YX: Funding acquisition, Resources, Visualization, Writing – review & editing. JW: Funding acquisition, Resources, Visualization, Writing – review & editing. PY: Funding acquisition, Resources, Visualization, Writing – review & editing.
